# LncRNA LINC01303 Promotes the Progression of Oral Squamous Cell Carcinomas via the miR-429/ZEB1/EMT Axis

**DOI:** 10.1155/2021/7974012

**Published:** 2021-12-06

**Authors:** Bo Sun, Xianyu Zheng, Weilong Ye, Pengcheng Zhao, Guowu Ma

**Affiliations:** ^1^School of Stomatology, Dalian Medical University, Dalian 116044, Liaoning, China; ^2^Department of Oral and Maxillofacial Surgery, The Second Affiliated Hospital of Dalian Medical University, Dalian 116027, Liaoning, China; ^3^College & Hospital of Stomatology, Anhui Medical University, Key Laboratory of Oral Diseases Research of Anhui Province, Hefei 230032, China

## Abstract

**Objectives:**

The aim of this research was to uncover the biological role and mechanisms of LINC01303 in oral squamous cell carcinoma (OSCC).

**Materials and Methods:**

Real-time quantitative PCR (qRT-PCR) was used to determine LINC01303 expression in OSCC tissues. Subcellular distribution of LINC01303 was examined by nuclear/cytoplasmic RNA fractionation and FISH experiments. The role of LINC01303 in the growth of TSCCA and SCC-25 was examined by CCK-8 assay, colony formation, transwell invasion assay in vitro, and xenograft tumor experiment in vivo. Dual-luciferase reporter assay was used to verify the interaction between LINC01303 and miR-429. RNA pull‐down assay was used to discover miR-429‐interacted protein, which was further examined by qRT-PCR, western blot, and rescue experiments.

**Results:**

LINC01303 expression was higher in OSCC tissues compared with adjacent nontumor tissues. LINC01303 was found to be localized in the cytoplasm of OSCC cells. Knockdown of LINC01303 inhibited OSCC cell proliferation and invasion, whereas increasing the expression of LINC01303 showed the opposite effects. Furthermore, LINC01303 served as a miR-429 “sponge” and positively regulated ZEB1 expression. Moreover, LINC01303 promoted OSCC through miR-429/ZEB1 axis both in vivo and in vitro.

**Conclusions:**

LINC01303 plays an oncogenic role in OSCC and is a promising biomarker for OSCC patients.

## 1. Introduction

Oral squamous cell carcinoma (OSCC) arises on the lip or oral cavity and always migrates to other parts of the body [1]. OSCC accounts for over 90% of all head and neck cancers with high incidences of associated morbidity and mortality worldwide [2, 3]. OSCC is characterized by local infiltration and metastasis of lymph nodes with poor prognosis and without a specific biomarker [4]; the five-year survival rate of OSCC is still very low despite the great improvement in clinical treatment [1, 5]. The development of OSCC is a multistep process that requires the combined effects of genetics and environment, that is, tobacco, alcohol, chronic inflammation, and viral infections [6, 7]. Although the identification of oncogenes and suppressors in OSCC has been performed in the past few decades, independent biomarkers that can be routinely applied to clinical treatment are lacking [8, 9]. Thus, discovering new indicators for OSCC patients is important.

Long noncoding RNAs (lncRNAs) are a class of noncoding RNA with lengths of over 200 nucleotides (nt); they cannot encode protein [10, 11]. LncRNAs are usually located in intergenic sequences, intronic sequences, or overlapping regions [12]. Currently, researchers discovered that lncRNAs could influence the expression of genes at multiple levels, such as transcriptional level [13, 14] and posttranscriptional level [15, 16]. There is growing evidence that lncRNAs act as suppressors or activators of mRNAs through competing with endogenous RNAs (ceRNAs) mechanisms [17]. In cancer, it is implied that lncRNAs play a vital role in the occurrence of human tumors by affecting cell activities [18, 19]. For example, lncRNA AC007271.3 can promote OSCC tumor cell growth and is a promising marker [20]. LINC01303 is conveniently located on the 14q23.1 chromosome; it is highly expressed in gastric cancer and inhibits tumor occurrence by mediating miR-101-3p [21]. LINC01303 is overexpressed in laryngeal squamous cell carcinoma (LSCC) patients and promotes LSCC by regulating miR-200c/TIMP2 axis [22]. In the present study, we observed that LINC01303 was highly expressed in OSCC patients, but the possible mechanisms remain unstated.

In our research, we found that LINC01303 was highly expressed in human OSCC tissues. The function and mechanism of LINC01303 were investigated. The knockdown of LINC01303 suppressed cell proliferation and invasion. High expression of LINC01303 markedly promoted OSCC. In addition, LINC01303 regulated miR-429/ZEB1/EMT axis by directly targeting miR-429 and enhancing ZEB1 expression. Our research uncovered that LINC01303 is a pivotal mediator in OSCC, and the LINC01303/miR-429/ZEB1/EMT axis may offer new clues for OSCC therapy.

## 2. Materials and Methods

### 2.1. Patient Tissue Samples

We collected OSCC tumor tissues from 50 cases along with matched adjacent normal tissues at Affiliated Stomatological Hospital of Anhui Medical University. All experiments were approved by the Research Scientific Ethics Committee of the Affiliated Stomatological Hospital of Anhui Medical University. All diagnosed patients signed an informed consent prior to the experiments.

### 2.2. Cell Culture

Normal human oral keratinocytes (NHOK) and OSCC cell lines (SCC-25, CAL-27, Tca8113, and TSCCA) were obtained from the Cell Bank of Type Culture Collection of Chinese Academy of Sciences (Shanghai, China). All cells were cultured in DMEM medium (Solarbio, Beijing, China) supplemented with 10% fetal bovine serum (FBS, Gibco, USA) and incubated in a cell incubator at 37°C and 5% CO_2_.

### 2.3. Isolation of Nuclear and Cytoplasmic RNA

Cytoplasmic and nuclear RNAs were isolated using P0028 Kit (Beyotime). Cells were rinsed with PBS, digested using trypsin, and collected after precipitation. We added 200 *μ*L of reagent A supplemented with RNase Inhibitor for every 20 *μ*L of cell precipitation. We added 20 *μ*L of reagent B and centrifuged the mixture at 12000 g for 5 min. We immediately transferred the supernatant to a precooled tube and added Trizol to extract the cytoplasmic RNA. In addition, the precipitation was collected, and Trizol was added to extract the nuclear RNA.

### 2.4. Fluorescence In Situ Hybridization (FISH) Assay

FISH assays were conducted using the Fluorescence In Situ Hybridization Kit (RiboBio, Guangzhou, Guangdong, China). We seeded cells on slides and fixed them in 4% paraformaldehyde (PFA) for half an hour under room temperature conditions. Then, 0.5% Triton X-100 on ice was used for 15 min to enhance membrane permeability. Subsequently, cells were mixed with hybridization buffer containing FISH probes for half an hour at 60°C. After washing off the residual reagent, the slides were dehydrated, and DAPI (Promega, Madison, WI, USA) was used for staining to observe the nucleus. The laser-scanning confocal microscope was used to observe the cells and obtain images (Leica Microsystems, Germany).

### 2.5. Cell Transfection

The specific small interfering RNAs against LINC01303 (si-LINC01303) and ZEB1 (si-ZEB1), scrambled siRNA control (si-NC), overexpressed plasmid (pcDNA3.1) LINC01303 (vector-LINC01303), and plasmid control (vector-NC) were synthesized by GenePharma Co., Ltd. (Shanghai, China). MiR-429 mimics (miR-429-mimics), mimic control (mimics-NC), miR-429 inhibitor (miR-429-inhibitor), and miRNA inhibitor negative control (inhibitor-NC) were synthesized by GenePharma Co., Ltd. Approximately 1 × 10^6^ cells were seeded in 6‐well plates with 2 mL complete medium. We routinely performed transfection with the help of Lipofectamine® 3000 (Invitrogen, U.S.A). qRT-PCR was used to detected the transfection efficiency 48 h after transfection.

### 2.6. RNA Isolation and RT‐qPCR Assay

RNA was isolated from normal and OSCC tissues and cells using TRIzol reagent (Thermo Fisher Scientific, Waltham, MA, USA) and then synthesized into cDNA by using the Prime Script™ 1st Strand cDNA Synthesis Kit (Takara, Tokyo, Japan). Subsequently, quantitative PCR was done by using SYBR Green reagent (Invitrogen) on the 7500 Fast Real-Time System (Invitrogen) to detect the RNA expression levels. The results were standardized to GAPDH or U6. The relative quantification of the indicated genes was normalized by using the 2^−ΔΔCt^ method. All indicators were assayed at least in triplicate. The primer information is listed in Table 1.

### 2.7. Cell Proliferation Assay

Cell viabilities were estimated by using the CCK‐8 detection kit (Apexbio, Houston, USA). Approximately 5 × 10^3^ treated TSCCA or SCC25 cells per well were cultured in 96-well plates and incubated for about 12 h. CCK‐8 solutions (10 *μ*L) were instilled per well 24, 48, 72, and 96 h later. Then, cells were incubated for another 4 h at 37°C. The absorbance value under 450 nm wavelength was detected by using a microplate reader (Thermo Fisher Scientific).

### 2.8. Colony Formation Assay

Approximately 1 × 10^3^ cells per well were cultured in a 6‐well plate and incubated in an incubator at 37°C and 5% CO_2_ for one week. Then, cells were washed twice in PBS, fixed in methanol for 30 min, and stained with 1% crystal violet dye. The number of colonies (a diameter ≥100 *μ*m) were counted. All assays were performed in triplicate.

### 2.9. Transwell Invasion Assay

As for transwell invasion assay, approximately 5 × 10^3^ treated TSCCA or SCC25 cells were resuspended and placed in the upper space of a transwell system (8 *μ*m pore size, Corning, USA) with a Matrigel-coated membrane (BD Bioscience, USA), which contained medium without serum. Lower chambers were supplemented with 100% complete culture medium. In consequence, hungry cells penetrated from the upper to the bottom and attached themselves to the membrane below. Afterward, the upper layer was removed, whereas the cells in the lower layer were retained for subsequent analysis. The number of invaded cells was determined by using 4% PFA to fix the retained cells and 0.1% crystal violet solution to dye the cells for 30 min. We randomly selected five mirror views to performed cell counts under a 200x microscope.

### 2.10. OSCC Tumor Xenograft In Vivo

Twenty-five male BALB/c nude mice (6 weeks old) were obtained from Shanghai SLRC Experimental Animal Center (Shanghai, China) and bred in a sterile environment with stable humidity and temperature. All animal procedures were approved by the Animal Research Ethics Committee of Affiliated Stomatological Hospital of Anhui Medical University. After transfection with sh-NC (*n* = 5), sh-LINC01303 (*n* = 5), vector-NC (*n* = 5), vector-LINC01303 (*n* = 5), or vector-LINC01303 + sh-ZEB1 (*n* = 5), approximately 1 × 107 TSCCA cells were diluted in 100 *μ*L medium and mixed well with a pipette. Then, they were hypodermically injected into the dorsal skin of nude mice. Tumor volume was measured by using a digital caliper on the 7th, 14th, 21st, and 28th day after injection and calculated using the following formula: tumor volume = 4*π*/3 × (width/2)^2^ × (length/2). When all procedures were completed, the nude mice were euthanatized, and tumor tissues were isolated for tumor weight examination.

### 2.11. Dual-Luciferase Reporter Assay

Wild-type LINC01303 (wt-LINC01303) or ZEB1 (wt-ZEB1) and mutant LINC01303 (mut-LINC01303) or ZEB1 (mut-ZEB1) were inserted into pGL3 vector (Promega). Subsequently, luciferase constructs and miR-429 mimics or negative control were cotransfected into cells with the help of Lipofectamine 3000 (Invitrogen). The Promega dual-luciferase assay system was used to evaluate efficiency at 48 h after the transfection.

### 2.12. RNA Pull‐Down Assay

Approximately 1 × 10^7^ TSCCA cells were harvested, lysed, and sonicated. The miR-429 probe (Bio-miR-429) and oligo probe (Bio-NC) were used for incubation with streptavidin magnetic beads (Life Technologies, U.S.A) at 25°C for 2 h to coat the beads with probes. The cell lysate and beads were incubated at 4°C for one night. The beads were cultured, and mRNA was extracted from the pull-down substance. qRT-PCR was used for analysis.

### 2.13. Western Blot Assay

Proteins were obtained by using pre‐cold RIPA buffer (Beyotime, Shanghai, China) with protease inhibitor. The concentration was determined with BCA assay, isolated by 12% SDS-PAGE, and transferred to PVDF membrane. The 5% nonfat milk was used to block nonspecific staining. Then, membranes were incubated with anti-GAPDH (Santa Cruz, C.A, 1 : 1000), anti-vimentin (Santa Cruz, C.A, 1 : 1000), anti-E-cadherin (Santa Cruz, C.A, 1 : 1000), anti-p-AKT (Santa Cruz, C.A, 1 : 1000), anti-AKT (Santa Cruz, C.A, 1 : 1000), and anti-ZEB1 (Santa Cruz, C.A, 1 : 1000) overnight at 4°C. After washing with PBST, membranes were incubated with secondary antibodies conjugated with HRP (Abcam, CA, U.S.A.) for 1 h at room temperature. The blots were visualized with ECL Plus Western blot detection reagent (Beyotime).

### 2.14. Statistical Analysis

GraphPad Prism 8.0 (GraphPad Software) was used for all statistical analyses. All experimental data were repeated thrice and presented as mean ± standard deviation. Student's *t*‐test was used to evaluate difference between two groups. The criterion of statistical significance was *P* < 0.05.

## 3. Results

### 3.1. LINC01303 Is Overexpressed in Human OSCC Tissues and Located in the Cytoplasm

**Figure 1:**
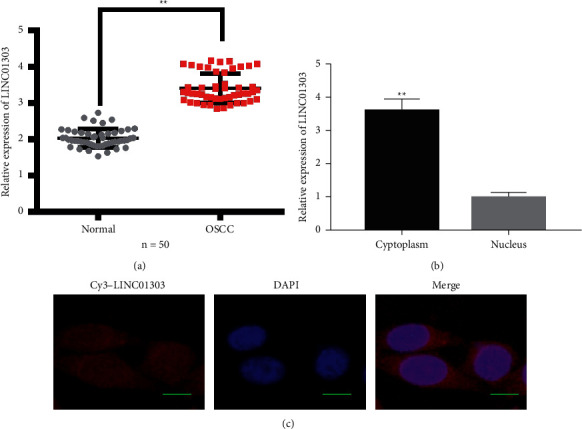
Identification and expression of LINC01303 in OSCC tissues and cells. (a) qRT-PCR detected the relative expression of LINC01303 in OSCC tumor tissues and their corresponding normal tissues. (b) Cell nuclear/cytoplasmic fractionation and qRT-PCR showed the cellular distribution of LINC01303 in TSCCA cells. (c) FISH analysis of LINC01303 in TSCCA cells. The nuclei were stained with DAPI, and LINC01303 was stained with red; scale bar: 10 *μ*m. ^*∗∗*^*P* < 0.01.

We used qRT-PCR analysis to examine the expression of LINC01303 in OSCC tumor tissues and their corresponding normal tissues to investigate the potential function of LINC01303 in OSCC. As shown in Figure 1(a), LINC01303 quantification was increased in OSCC tissues. In addition, we discovered that LINC01303 is located in the cytoplasm (Figure 1(b)). Moreover, as shown in Figure 1(c), LINC01303 was distributed in the cytoplasm of OSCC cells, as found through FISH assay.

### 3.2. LINC01303 Promotes OSCC Cell Proliferation, Migration, and Invasion In Vitro

**Figure 2:**
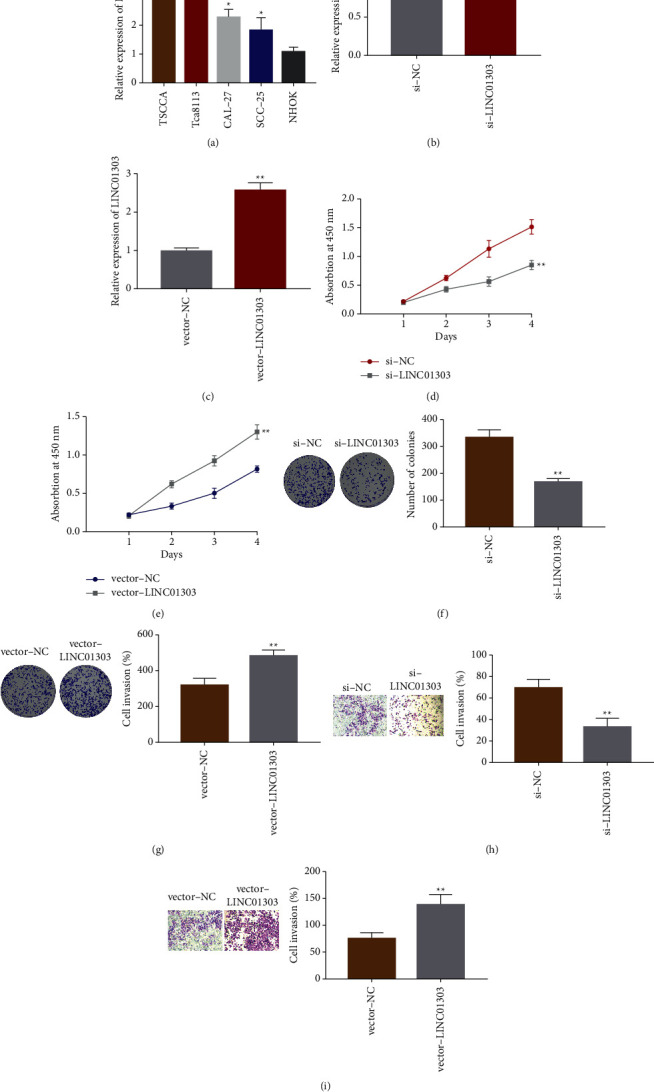
LINC01303 promoted OSCC cell proliferation, migration, and invasion in vitro. (a) qRT-PCR analysis was performed to examine the expression of LINC01303 in OSCC cell lines (TSCCA, Tca8113, CAL-27, and SCC-25) and NHOK cell. (b) qRT-PCR analysis was employed to examine the LINC01303 expression in TSCCA cells transfected with siRNA LINC01303. (c) qRT-PCR was employed to measure the LINC01303 expression in TSCCA cells transfected with LINC01303 overexpressed plasmid. CCK-8 assay was used to investigate the proliferation of TSCCA cells transfected with siRNA LINC01303 (d) and SCC-25 cells transfected with LINC01303 overexpressed plasmid (e). Colony formation assays of TSCCA cells transfected with siRNA LINC01303 (f) and SCC-25 cells transfected with LINC01303 overexpressed plasmid (g). Transwell assay was used to test the cell invasion of TSCCA cells transfected with siRNA LINC01303 (h), and SCC-25 cells transfected with LINC01303 overexpressed plasmid (i). ^*∗*^*P* < 0.05, ^*∗∗*^*P* < 0.01.

The effects of LINC01303 on OSCC cell proliferation, migration, and invasion were tested. First, we quantified LINC01303 in four OSCC and NHOK cell lines and found that four OSCC cells (especially TSCCA and Tca8113) showed higher quantification of LINC01303 than NHOK cell (Figure 2(a)). Then, we silenced LINC01303 via siRNA in TSCCA cell line (Figure 2(b)). Knockdown of LINC01303 in TSCCA cell line reduced cell reproductive capacities over a four-day culture (Figure 2(d)). In accordance with the previously mentioned results, silencing LINC01303 by siRNA also markedly reduced cell colony numbers and invaded cell numbers of the TSCCA cell line (Figures 2(f) and 2(h)). On the contrary, we detected the upregulation of LINC01303 in SCC-25 cell line transfected with LINC01303 overexpressed plasmid (Figure 2(c)). Overexpression of LINC01303 in SCC-25 cell line led to elevated cell reproductive capacities over a four-day culture (Figure 2(e)). In accordance with this result, upregulating LINC01303 by plasmid also increased the cell colony numbers and invaded cell numbers of SCC-25 cell line (Figures 2(g) and 2(i)).

### 3.3. LINC01303 Promotes OSCC Tumor Growth and Migration In Vivo

**Figure 3:**
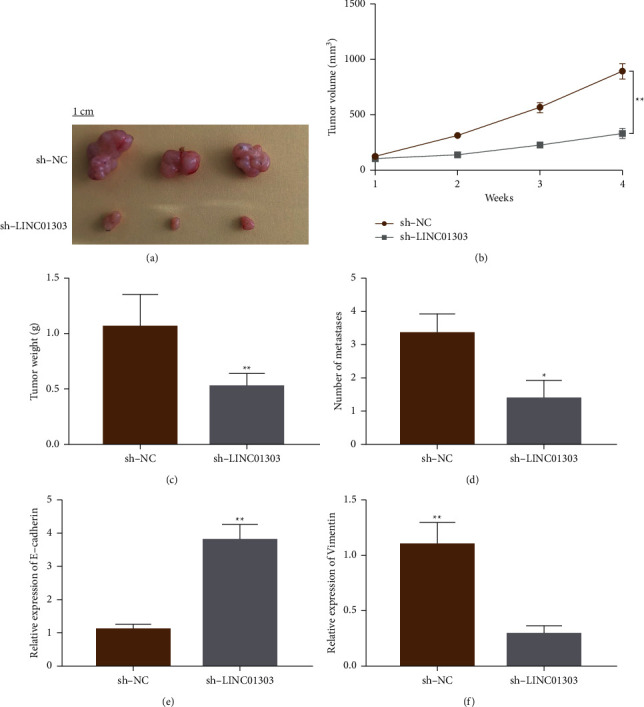
LINC01303 promotes OSCC cell growth and migration in vivo. (a) Images of tumor formation in nude mice (*n* = 5) injected subcutaneously with TSCCA cells with knockdown of LINC01303 (bottom) or the control (top). Effect of LINC01303 knockdown on tumor volume (b) and weight (c) of xenografts derived from TSCCA cells. (d) Number of metastatic foci detected by H&E staining induced by LINC01303 knockdown. mRNA relative expression of E-cadherin (e) and vimentin (f) in nude mice tumors tissues. ^*∗*^*P* < 0.05; ^*∗∗*^*P* < 0.01.

Furthermore, to determine the effects of LINC01303 on OSCC tumor growth in vivo, LINC01303-knockdown (sh-LINC01303) or control (sh-NC) TSCCA cells were injected subcutaneously into nude mice. The tumor volume and weight in mice injected with sh-LINC01303 TSCCA cells were notably reduced compared with the previously mentioned index in control mice (Figures 3(a)–3(c)). In agreement with these data, a decreased level of metastases in LINC01303-depleted tumors was observed compared with the control tumors (Figure 3(d)). We used qRT-PCR to quantify E-cadherin and vimentin in tumors. Consistent with the finding that LINC01303 silencing inhibited tumor metastasis in vivo, E-cadherin expression was upregulated in the sh-LINC01303 group, whereas vimentin was downregulated (Figures 3(e) and 3(f)).

### 3.4. LINC01303 Acts as miR-429 Sponge

**Figure 4:**
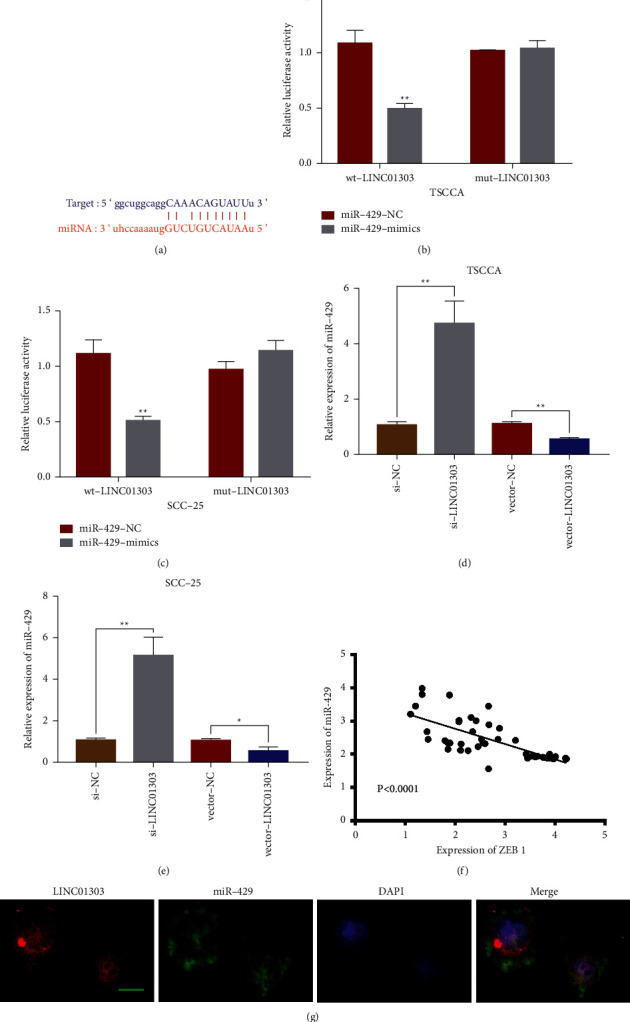
LINC01303 directly targeted miR‐429. (a) starBase prediction of LINC01303 and miR-429 sites. Dual luciferase reporter analysis of the targeted relationship between LINC01303 and miR-429 in TSCCA cells (b) and SCC-25 cells (c). qRT‐PCR was used to examine the expression level of miR‐429 after transfection with si‐NC, si‐LINC01303, vector-NC, or vector-LINC01303 in TSCCA cells (d) and SCC-25 cells (e). (f) The linear correlations of LINC01303 and miR-429 expression were demonstrated by Pearson's analysis. (g) FISH analysis was used to detect the colocalization of LINC01303 and miR-429 in TSCCA cells. The nuclei were stained with DAPI (blue), LINC01303 was stained with Cys (red), and miR-429 was stained with FAM (green). The red arrow shows colocalization of LINC01303 and miR-429. Scale bar: 10 *μ*m. ^*∗∗*^*P* < 0.01.

Previous studies supported the finding that lncRNA served as miRNA “sponge.” We predicted the putative miRNAs of LINC01303 through starBase [23] online website (https://starbase.sysu.edu.cn/) and found a targeted relationship between miR-429 and LINC01303 (Figure 4(a)). The dual-luciferase reporter assay showed that miR-429 could inhibit the luciferase activity of wt-LINC01303 in TSCCA and SCC-25 cell lines (Figures 4(b) and 4(c)). Moreover, miR-429 relative expression increased notably in TSCCA and SCC-25 cell lines after transfection with si-LINC01303. Consistent with these data, miR-429 relative expression in TSCCA and SCC-25 cell lines after transfection with vector-LINC01303 decreased notably (Figures 4(d) and 4(e)). Moreover, Pearson's correlation analysis suggested that LINC01303 quantification was significantly and negatively correlated with miR-429 expression in OSCC (Figure 4(f)). Furthermore, RNA FISH assay revealed that LINC01303 and miR-429 were colocalized in the cytoplasm of TSCCA cell (Figure 4(g)). The previously mentioned results uncovered that LINC01303 could directly target miR-429.

### 3.5. ZEB1 Was a Direct Target of miR-429

**Figure 5:**
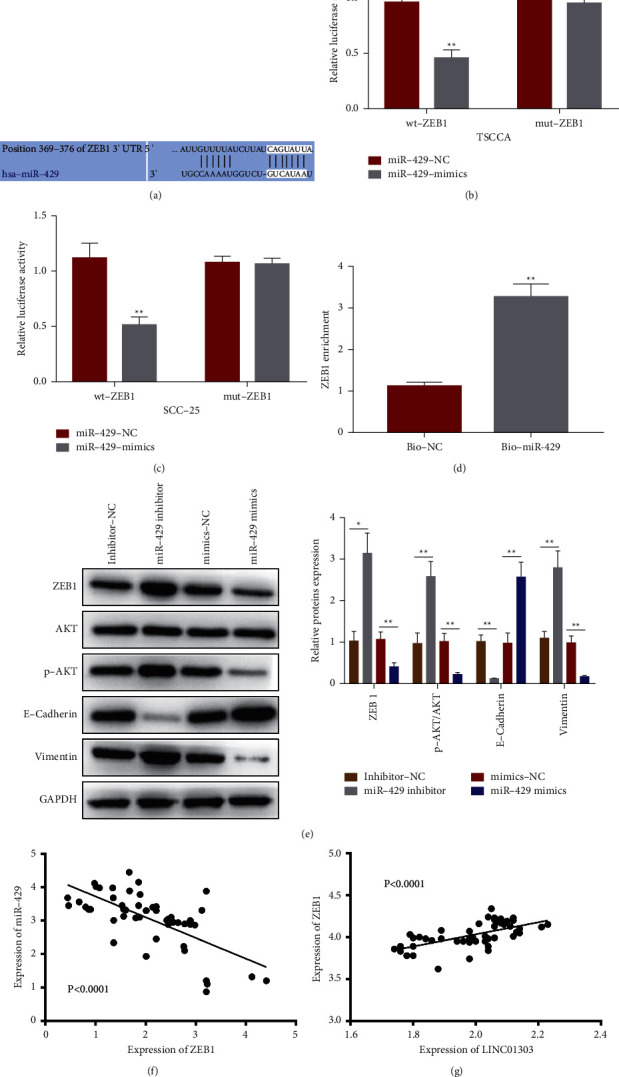
ZEB1 was targeted by miR‐429 in OSCC. (a) ZEB1 was the putative target of miR‐429 predicted by TargetScan. Luciferase reporter analysis of the targeted BINDING between ZEB1 and miR-429 in TSCCA cells (b) and SCC-25 cells (c). (d) RNA pull-down assay to detect the binding of ZEB1 and miR-429. (e) Western blot was used to detect the expression levels of ZEB1, AKT, p-AKT, E-cadherin, and vimentin after transfection with inhibitor-NC, miR-429 inhibitor, mimics-NC, and miR-429 mimics in TSCCA cells. The linear correlations of miR-429 and ZEB1 (f) or ZEB1 and LINC01303 (g) expression were demonstrated by Pearson's analysis. ^*∗*^*P* < 0.05; ^*∗∗*^*P* < 0.01.

Through the TargetScan [24] database, we found that ZEB1 is a potential target of miR-429.  Figure 5(a) shows their matching binding sites. The luciferase reporter assay shows that wild-type (wt) ZEB1 could change the relative luciferase activity in TSCCA and SCC-25 cells, whereas no obvious change in the luciferase activity was detected in the mut 3′-UTR of ZEB1 (Figures 5(b) and 5(c)), thereby indicating the direct interaction between ZEB1 and miR-429. We next used biotinylated miR‐429 probe (Bio-miR-429) to pull down ZEB1 mRNA. The endogenous ZEB1 was enriched specifically in Bio-miR‐429 probe compared with Bio-NC, indicating that ZEB1 was a direct negative target of miR-429 (Figure 5(d)). We performed immunoblotting assay to explore the mechanisms mediated by miR-429 in TSCCA cell. The increasing quantification of miR‐429 attenuated the protein expressions of ZEB1, p-AKT, and vimentin and improved the protein expression of E-cadherin (Figure 5(e)). Conversely, ZEB1, p-AKT, and vimentin expressions were improved in miR‐429 inhibitor-transfected TSCCA cells. Furthermore, the correlation analysis showed that miR-429 quantification was inversely and significantly correlated with ZEB1 in OSCC (Figure 5(f)). ZEB1 quantification was positively and significantly correlated with LINC01303 in OSCC (Figure 5(g)). Thus, miR‐429 negatively regulated the quantification of ZEB1 by directly targeting the 3′-UTR regions of ZEB1 in TSCCA cells.

### 3.6. LINC01303 Mediates miR-429/ZEB1 Axis to Promote OSCC

**Figure 6:**
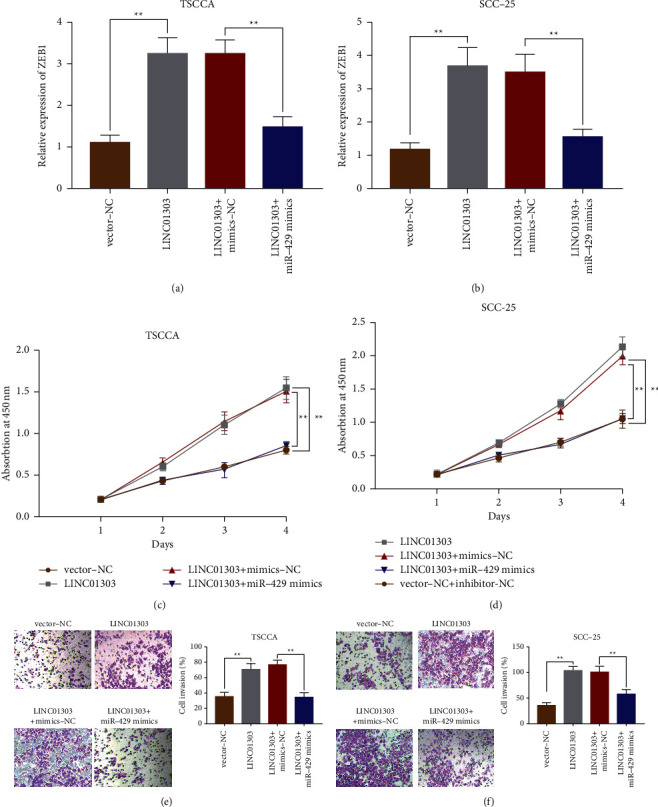
LINC01303 promote OSCC cell proliferation and invasion via miR-429/ZEB1 axis. qRT‐PCR was employed to test the expression of ZEB1 after cotransfection with sh-NC + inhibitor-NC, sh-NC + miR-429 inhibitor, sh-LINC01303 + inhibitor-NC, or sh-LINC01303 + miR-429 inhibitor in TSCCA cells (a) and SCC-25 cells (b). CCK-8 assay was employed to test cell proliferation abilities after cotransfection in TSCCA cells (c) and SCC-25 cells (d) of the previously mentioned groups. Transwell assay was employed to test cell invasion after cotransfection in TSCCA cells (e) and SCC-25 cells (f) of the previously mentioned groups. ^*∗∗*^*P* < 0.01.

To confirm that LINC01303 participated in tumor developmental process through miR-429/ZEB1 axis, we carried out cotransfection experiments. TSCCA and SCC-25 cells were transfected with sh-NC + inhibitor-NC, sh-NC + miR-429 inhibitor, sh-LINC01303 + inhibitor-NC, and sh-LINC01303 + miR-429 inhibitor. We determined the ZEB1 expression, proliferation, and invasion through experiments. qRT-PCR results showed that ZEB1's relative expression in TSCCA and SCC-25 cell lines increased notably after transfection with sh-NC + miR-429 inhibitor. However, the upregulation of ZEB1 mediated by miR-429 inhibitor transfection was inhibited by sh-LINC01303 + miR-429 inhibitor co-transfection (Figures 6(a) and 6(b)). According to CCK-8 experiment, OSCC cells (TSCCA and SCC-25) proliferation abilities were obviously enhanced after the transfection with sh-NC + miR-429 inhibitor (Figures 6(c) and 6(d)). Transwell experiment indicated that the invaded cell numbers of OSCC were increased after transfection with sh-NC + miR-429 inhibitor (Figures 6(e) and 6(f)). However, cell proliferation and invasion activities of OSCC cells (TSCCA and SCC-25) were significantly decreased after cotransfection with sh-LINC01303 with miR-429 inhibitor (Figures 6(c)–6(f)), which indicated that LINC01303 mediated miR-429/ZEB1 axis to promote OSCC.

### 3.7. Downregulation of ZEB1 Reverses Tumor Phenotype Mediated by LINC01303 Overexpression In Vitro and In Vivo

**Figure 7:**
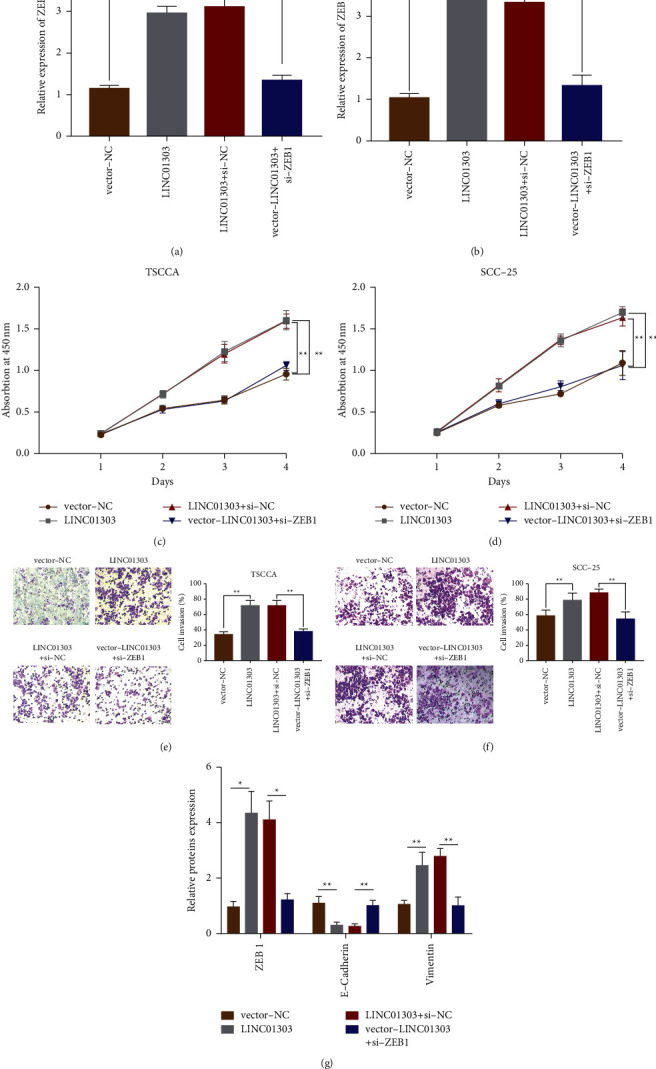
Downregulation of ZEB1 reverses the tumor phenotype induced by LINC01303 overexpression in vitro. qRT‐PCR was employed to test ZEB1 mRNA relative expression after cotransfection with vector-NC + si-NC, vector-NC + si-ZEB1, vector-LINC01303 + si-NC, or vector-LINC01303 + si-ZEB1 in TSCCA cells (a) and SCC-25 cells (b). CCK-8 assay was employed to test cell proliferation abilities after cotransfection in TSCCA cells (c) and SCC-25 cells (d) of the previously mentioned groups. Transwell assay was employed to test cell invasion after cotransfection in TSCCA cells (e) and SCC-25 cells (f) of the previously mentioned groups. (g) Western blot was employed to test the expression levels of ZEB1, E-cadherin, and vimentin after cotransfection in TSCCA cells of the previously mentioned groups. ^*∗*^*P* < 0.05, ^*∗∗*^*P* < 0.01.

We examined the potential biological mechanisms underlying ZEB1's involvement in OSCC cell activities. We carried out cotransfection experiments. TSCCA and SCC-25 cells were transfected with vector-NC + si-NC, vector-NC + si-ZEB1, vector-LINC01303 + si-NC, and vector-LINC01303 + si-ZEB1. We determined ZEB1 expression, proliferation, and invasion through experiments. qRT-PCR results uncovered that ZEB1 relative quantification in TSCCA and SCC-25 cell lines increased after transfection with vector-LINC01303 + si-NC. However, the upregulation of ZEB1 mediated by vector-LINC01303 transfection was inhibited by vector-LINC01303 + si-ZEB1 cotransfection (Figures 7(a) and 7(b)). According to the results of the CCK-8 experiment, OSCC cell (TSCCA and SCC-25) proliferation abilities were obviously enhanced after transfection with vector-LINC01303 + si-NC (Figures 7(c) and 7(d)). Transwell experiment uncovered that OSCC cell invasion abilities were reinforced after transfection with vector-LINC01303 + si-NC (Figures 7(e) and 7(f)). However, cell proliferation and invasion activities of OSCC cells (TSCCA and SCC-25) were significantly decreased after cotransfection with vector-LINC01303 + si-ZEB1 (Figures 7(c)–7(f)), which indicated that ZEB1 reversed the OSCC cell proliferation and invasion mediated by LINC01303 overexpression. We performed immunoblotting assay to explore the mechanisms mediated by ZEB1 in TSCCA cell. The enhanced quantification of LINC01303 increased the protein quantification of vimentin and decreased the protein expression of E-cadherin (Figure 7(g)). Conversely, vimentin expression was distinctly inhibited, and E-cadherin expression improved in vector-LINC01303 + si-ZEB1-transfected TSCCA cells.

## 4. Discussion

The OSCC development is a multistage process that includes a variety of changes in gene expression levels and signal transduction pathways. Effective tumor markers can reduce tumor recurrence rate and increase survival rate; finding such markers could provide important guidance for clinical treatment [25]. Even though many new OSCC biomarkers have been identified in recent years [26–28], their mechanisms remain unclear. As an emerging tumor biomarker, LINC01303 has been extensively studied, but its role in OSCC is unknown. In the current research, we assessed the expression levels of LINC01303 in OSCC patients and controls to find out whether LINC01303 can serve as a promising biomarker for the early detection and prediction of OSCC.

LncRNAs have functions in many cancers. Researchers uncovered that the expressions of LncRNAs, UCA1 [29], CASC9 [30], and FAL1 [31] promote tumor progression in OSCC. Furthermore, Gong and Maquat [15] indicated a relationship between LINC01303 and gastric cancer [21] or laryngeal squamous cell carcinoma [22]. Whether LINC01303 serves a function in the tumor progression of OSCC has not been reported. The current research disclosed that LINC01303 was a mediator in OSCC tumorigenesis. We discovered the biological mechanisms underlying LINC01303's influence on cell activities of OSCC. We observed that increasing the expression of LINC01303 could reinforce the cell proliferation and invasion of OSCC cells through the downregulation of miR-429.

The miR-429 is part of the miR-200 family. Increasing evidences have disclosed that the dysregulation of miR-429 is involved in epithelial-mesenchymal transition (EMT), progression, invasion, and metastasis of a variety of cancers. MiR-429 has a tumor-promotion role in some cancers. However, miR-429 appears to have a tumor-suppressing role in many cancers, including breast cancer [32], bladder cancer [33], and thyroid cancer [34]. The inhibition of miR-429 could suppress the proliferation, migration, and invasion of cancer cells. Additionally, a relationship was found between miR-200 family and OSCC [35]. Lei et al. [36] revealed that miR-429 inhibits OSCC tumor growth by targeting zinc finger E-box binding homeobox 1 (ZEB1). Researchers have revealed that ZEB1 could be used as a biomarker for the progression and recurrence of OSCC patients; targeting ZEB1 may provide novel strategies for the treatment of OSCC patients [37]. In this research, we uncovered that miR-429 regulated ZEB1 gene expression by targeting its 3′-UTR regions in OSCC.

EMT is a complicated process and is considered to be a commitment step in tumor occurrence [38]. ZEB1 negatively regulated E-cadherin expression and was significantly involved in EMT induction [39–41]. In addition, undergoing EMT usually leads to the loss of E-cadherin expression and increase in vimentin expression in metastatic tumor tissues [42]. Herein, we examined the changes of E-cadherin and vimentin and evaluated the functional role of ZEB1 in OSCC tumorigenesis. We found that the upregulation of LINC01303 inhibited E-cadherin and promoted vimentin, thereby implying the occurrence of the EMT process. In addition, ZEB1 expression was positively correlated with LINC01303, and its downregulation reversed tumor phenotype mediated by LINC01303 overexpression in vitro and in vivo. In summary, LINC01303 promoted OSCC progression through miR-429/ZEB1/EMT axis. The main limitation of the present work is the use of a heterotopic xenotransplantation in the subcutis. The role of LINC01303 in OSCC can be better evaluated by using an in situ tumor model. This study may provide new diagnostic markers and new drug therapeutic targets for OSCC treatment.

In conclusion, LINC01303 was upregulated in OSCC tissues and cells. LINC01303 might act as a “sponge” of miR-429 and be prominently conducive to OSCC occurrence by activating ZEB1 protein levels. The LINC01303/miR‐429/ZEB1/EMT axis could provide more effective clues for the clinical treatment of OSCC patients.

## Figures and Tables

**Table 1:** Primer sequences.

Gene	Forward sequence (5′-3′)	Reverse sequence (5′-3′)
GAPDH	TCAAGGCTGAGAACGGGAAG	TGGACTCCACGACGTACTCA
U6	CTCGCTTCGGCAGCACATATACT	CGCTTCACGAATTTGCGTGT
ZEB1	CAGCTTGATACCTGTGAATGGG	TATCTGTGGTCGTGTGGGACT
miR-429 mimics	TGCCAAAATGGTCTGTCATAAT	ACGGTTTTACCAGACAGTATTA

miR-429 inhibitor	ATTATGACAGACCATTTTGGCA	TAATACTGTCTGGTAAAACCGT
miR-429 NC	ATTACTAGGCTATGCATGCTAG	TAATGATCCGATACGTACGATC

## Data Availability

The analysed datasets generated during the study are available from the corresponding author on reasonable request.
